# PSMA antibody, humanized PSMA.CAR10.3, or Cetuximab increases prostate cancer localization of NF-κB p50-deficient immature myeloid cells (p50-IMC) and phagocytosis by their macrophage progeny

**DOI:** 10.1007/s00262-024-03939-4

**Published:** 2025-02-04

**Authors:** Mohammad A. Alzubi, Theresa Barberi, Alan D. Friedman

**Affiliations:** Division of Pediatric Oncology, Department of Oncology, Johns Hopkins University, CRB I, Rm. 253, 1650 Orleans St., Baltimore, MD 21231 USA

**Keywords:** Myeloid, Immunotherapy, NF-κB p50, Prostate cancer, PSMA, EGFR

## Abstract

Adoptive transfer of immature myeloid cells lacking the repressive NF-κB p50 subunit (p50-IMC) slows the growth of syngeneic murine prostate cancer and other tumors. Directing p50-IMC to tumors using Fc receptor-bound antibodies (Abs) or surface chimeric antigen receptors (CARs) may increase tumor localization and subsequent phagocytosis of cancer cells by their mature myeloid progeny, potentiating anti-tumor T cell activation. PSMA and EGFR are found on aggressive human prostate cancers, and p50-IMC express receptors that bind the antibody Fc domain. p50-IMC combined with PSMA Ab, EGFR Ab (Cetuximab), or fully humanized PSMA.CAR10.3 manifest increased localization to Myc-CaP murine prostate cancer tumors expressing PSMA or EGFR. Tumor localization is further increased when myelo-depleting 5-fluorouracil precedes p50-IMC administration. Additionally, we find that PSMA Ab, EGFR Ab, or PSMA.CAR10.3 increase in vitro phagocytosis of Myc-CaP cells expressing PSMA or EGFR by p50-IMC-derived macrophages, including in M2-promoting IL-4, which is a component of the immune-suppressive tumor microenvironment. Lack of tolerance of human PSMA or EGFR by immune-competent mice and lack of expression of human PSMA protein in the prostate of AR_2_-Probasin-hPSMA transgenic mice precluded our ability to determine whether human-specific PSMA or EGFR antibody or PSMA.CAR10.3 increases anti-tumor efficacy of murine p50-IMC. Nevertheless, this study indicates the potential clinical utility of adding a tumor-directing antibody or CAR, including the novel, fully humanized PSMA.CAR10.3, to proinflammatory p50-IMC to optimize the activation of anti-tumor immunity in prostate cancer and other malignancies, and understanding PSMA toxicity in normal but not malignant prostate epithelium may reveal a novel therapeutic opportunity.

**Supplementary Information:**

The online version contains supplementary material available at 10.1007/s00262-024-03939-4.

## Introduction

Metastatic, castration-resistant prostate cancer (mCRPC) is uniformly fatal, with 5-year survival only ~ 30%. Neither PD-1 nor CTLA-4 antibody (Ab) therapy leads to response in the vast majority of individuals with mCRPC [1]. Many solid tumors harbor abundant M2 macrophages, which impair effector T cell function. In contrast, proinflammatory M1 macrophages enhance anti-tumor immunity [2]. Prostate cancer progression is associated with increased numbers of M2 macrophages [3–5].

Myeloid cells lacking intact *Nfkb1* alleles encoding the repressive NF-κB p50 (p50) subunit exhibit a proinflammatory phenotype [6, 7]. Prostate cancer and additional malignancies grow slower in p50^−/−^ mice or in p50^f/f^;LysM-Cre mice lacking p50 specifically in myeloid cells, associated with increased total and activated tumor T cells [8–12].

In the immune-competent B6CaP murine prostate cancer model, adoptive transfer of syngeneic immature myeloid cells lacking NF-κB p50 (p50-IMC), administered after a single dose of 5-fluorouracil (5-FU), slows tumor growth > threefold, whereas 5-FU followed by immature myeloid cells from wild-type mice, 5-FU alone, or p50-IMC alone are ineffective [8]. In contrast to mature macrophages, which largely localize to the liver upon adoptive transfer [13], IMC more effectively reach tumors [8]. p50-IMC develop into macrophages and dendritic cells, and although mature myeloid progeny of p50-IMC comprise only ~ 2% of tumor myeloid cells after a single injection, after three p50-IMC injections tumor CD8^+^ T cell numbers increase fourfold and the proportion of CD8^+^ T cells that express IFNγ increases twofold. Further, CD8^+^ T cell depletion eliminates the ability of 5-FU/p50-IMC to slow prostate cancer growth [8]. 5-FU/p50-IMC immunotherapy also slows the growth of syngeneic murine pancreatic ductal carcinoma [8], neuroblastoma [12] and glioblastoma (T.B. and A.D.F., unpublished). Although 5FU/p50-IMC immunotherapy slows tumor progression, it is not curative.

Prostate-specific membrane antigen (PSMA) levels often increase steadily during malignant prostate cancer progression, including after ADT [14–16]. Epidermal growth factor receptor (EGFR) is expressed on 41% of newly diagnosed prostate cancers, 76% of CRPCs, and 100% of metastatic prostate cancers [17]. p50-IMC and their myeloid progeny express receptors that bind the Ab Fc domain. Herein, we demonstrate that anti-human PSMA or EGFR Abs increase in vitro phagocytosis of Myc-CaP (MC) prostate cancer cells expressing human PSMA or EGFR (hPSMA, hEGFR) by macrophages derived from murine p50-IMC. Further, using immune-deficient NSG mice, we observe that these same Abs increase in vivo localization to tumors derived from these cell lines. In addition, we developed a novel, fully humanized chimeric antigen receptor specific for human PSMA (PMSA.CAR10.3), which, when expressed on p50-IMC, also increases phagocytosis of MC/hPSMA cells and localization to MC/hPSMA tumors.

We found that immune-competent mice do not tolerate hPSMA or hEGFR. Additionally, an (AR)_2_-Probasin promoter-hPSMA transgenic line only expresses hPSMA RNA, but not protein, in the prostate and retains intolerance to syngeneic MC/hPSMA cells. These limitations precluded evaluation of whether human-specific PSMA Ab or PSMA.CAR10.3 increase p50-IMC efficacy in an immune-competent setting. Nevertheless, our findings indicate that combining Abs or CARs with human p50-IMC may increase their efficacy against prostate cancer and other malignancies.

## Materials and methods

### Plasmids construction

The human PSMA (hPSMA), hPSMA(NΔ9) [18] and human EGFR (hEGFR) cDNAs were subcloned into the MIPuro retroviral vector. The PSMA.CAR10.3 cDNA was assembled by combining synthetic DNA segments (Blue Heron Biotech, Bothell, WA, USA) that encode a human IgG_1_ leader peptide, V_H_ and V_L_ domains from PSMA Ab10.3 [19] linked by a (GGGGS)_3_ peptide, spacer and trans-membrane domains from hCD8, and an intracellular domain from hCD3ζ, and then inserted into MIPuro. PSMA Ab10.3 was generated using a mouse that only contains human immunoglobulin genes [20]. The AR_2_-Probasin promoter (Pbn) regulatory elements used to generate Hi-Myc mice [21] were positioned upstream of the hPSMA cDNA and an SV40 splice and polyA site from p19Luc [22]. AR_2_-Pbn-hPSMA-polyA released from vector DNA was microinjected into FVB/N blastocysts by the Johns Hopkins Transgenic Core, and founders were identified by tail DNA PCR.

### Cell culture

MC (ATCC, CRL-3255, Manassas, VA, USA), TRAMP-C1 (TC1, ATTC, CRL 2730), and 293 T (ATCC, CRL-3216) cells were maintained in DMEM with 10% heat-inactivated (HI)-FBS and antibiotic/antimycotic (AA, Sigma, Burlington, MA, USA). LNCaP cells (ATCC, CRL-1740) were cultured in RPMI with 10% HI-FBS and AA. Retroviral vectors were packaged by transfection with pkat2ecopac into 293 T cells using Lipofectamine 2000 [23]. MC and TC1 cells were transduced using 293 T supernatant and Polybrene (4 μg/mL), followed by puromycin selection (6 μg/mL) and flow-sorting of hPSMA- or hEGFR-expressing cells. IMC were generated as described [8]. M1- or M2-polarized macrophages were generated by culturing IMC with IMDM, 10% HI-FBS, AA, and murine M-CSF (10 ng/mL) on six-well dishes (1E6 cells/well) for six days, followed by removal of M-CSF and addition of murine IFNγ (50 ng/mL) or murine IL-4 (10 ng/mL) for 24 h, respectively. Cytokines were from Peprotech (Cranbury, NJ, USA). IMC were transduced by spinoculation with Polybrene in 12-well dishes (1500 × g for 2 h at 22 °C), culture at 37 °C for 4 h, then washed and resuspended in expansion media, followed two days later by puromycin selection (2 μg/mL) for 48 h and then continued expansion.

### Phagocytosis

For phagocytosis assays, MC lines were labeled with CFSE by incubating cells in phosphate-buffered saline (PBS), 5% HI-FBS, 5 μM CFSE (Invitrogen, Carlsbad, CA, USA) at 37 °C for 10 min, followed by washing with DMEM/10% HI-FBS, and culture at 37 °C with DMEM/10% HI-FBS, AA for 45 min in an ultra-low attachment (ULA) plate. For Ab-mediated phagocytosis, CFSE-labeled MC lines were incubated for 30 min at 4 °C in PBS, 0.5% BSA, 2 mM EDTA with 10 μg/mL PSMA Ab3.9, EGFR Ab (Cetuximab), or isotype control—murine IgG2b or human IgG1 (Bio-X-Cell, Lebanon, NH, USA), respectively—followed by washing and resuspension in 4 °C IMDM at 1E6 cells/mL. Purified PSMA Ab3.9 was generated from a hybridoma line (ATCC, PTA-3258) by Bio-X-Cell. For CAR-mediated phagocytosis, macrophages were generated from MIPuro- or MIPuro-PSMA.CAR10.3-transduced IMC. Macrophages were released from culture dishes using TrypLE, washed and resuspended in 4 °C IMDM at 1E6 cells/mL. 5E4 macrophages were added to 1E5 CFSE-labeled MC cells in round-bottomed ULA 96 well plates and cultured for 3 h at 37 °C, followed by flow cytometry for CFSE and CD11b. In some experiments, MC lines labeled using 0.2 μM pHrodo, Red SE (Invitrogen) in PBS were co-cultured for 3 h with macrophages labeled with CFSE, followed by phase contrast and green (FITC channel) and red (TRITC channel) fluorescence imaging using an Eclipse Ti-U microscope (Nikon, Melville, NY, USA).

### Mice and tumor localization

WT C57BL/6 (B6) and FVB/N mice were from Charles River Laboratories (Wilmington, MA, USA). B6 p50^−/−^ (#006097) and NSG (#005557) mice were from Jackson Laboratory (Bar Harbor, ME, USA). Eight- to sixteen-week-old male mice were utilized. For MC lines, 1E6 cells in 100 μL Hanks’ balanced salt solution (HBSS) or 200 μL Matrigel (#354234, Corning, Corning, NY, USA):HBSS (1:1) were inoculated subcutaneously into the mouse flank, and tumor sizes were monitored using calipers. Tumor volumes were estimated as length x width-squared divided by two. IMC were labeled by incubating cells in HBSS, 5 μM CFSE at 37 °C for 10 min, followed by washing and culture with DMEM/10% HI-FBS, AA for 45 min in an ULA plate. 1E7 CFSE-labeled IMC were injected into tumor-bearing NSG mice via retroorbital injection. 5-FU (112.5 mg/kg) was administered intraperitoneally five days prior to injection. For Ab-mediated localization, IMC were incubated for 30 min at 4 °C in HBSS with 100 μg Ab or isotype control just prior to injection. Tumors were isolated 24 h later, dissociated into single cells as described [14], and then subjected to flow cytometry for viability, CFSE, and CD11b.

### Flow cytometry

Cell staining was conducted on ice in PBS with 3% HI-FBS and 5 mM EDTA. After block with FcγR2/FcγR3 Ab (except when staining for FcγRs) for 15 min, specific Abs were added for 45 min. Cells were analyzed using an LSR Fortessa Flow Cytometer (BD Biosciences, Menlo Park, CA, USA), gating on cells that exclude Live/Dead Aqua (Invitrogen). Antibodies used were: anti-CD11b-APC, anti-CD11b-AlexaFluor-647 (Alexa-647), anti-hPSMA-APC, anti-hEGFR-Alexa-647, PSMA Ab3.9 with goat anti-murine IgG-PE, Cetuximab or PSMA Ab10.3 with rat anti-human IgG-PE, anti-FcγR2-APC, anti-FcγR3-PE-Cy7, and anti-FcγR4-BV421. Labeled Abs were from BioLegend (San Diego, CA, USA). PSMA.CAR10.3 expression and interaction with hPSMA was evaluated using hPSMA-biotin (ACROBiosystems, Newark, DE, USA) and streptavidin (SA)-APC. CFSE was detected in the FITC channel.

### Protein and RNA analysis

Total cellular proteins in Laemmli sample buffer were subjected to Western blotting using anti-murine/human PSMA Ab (#12815, Cell Signaling Technology, Danvers, MA, USA) and *β*-actin Ab (AC-15, Sigma) as described [23]. RNA was quantified using real-time PCR (qRT-PCR) as described [10].

### Data analysis

Phagocytosis and tumor localization values were compared using the Student *t* test. Means and SD values are shown.

## Results

### Murine prostate cancer lines expressing human PSMA or EGFR and cognate antibodies

**Fig. 1:**
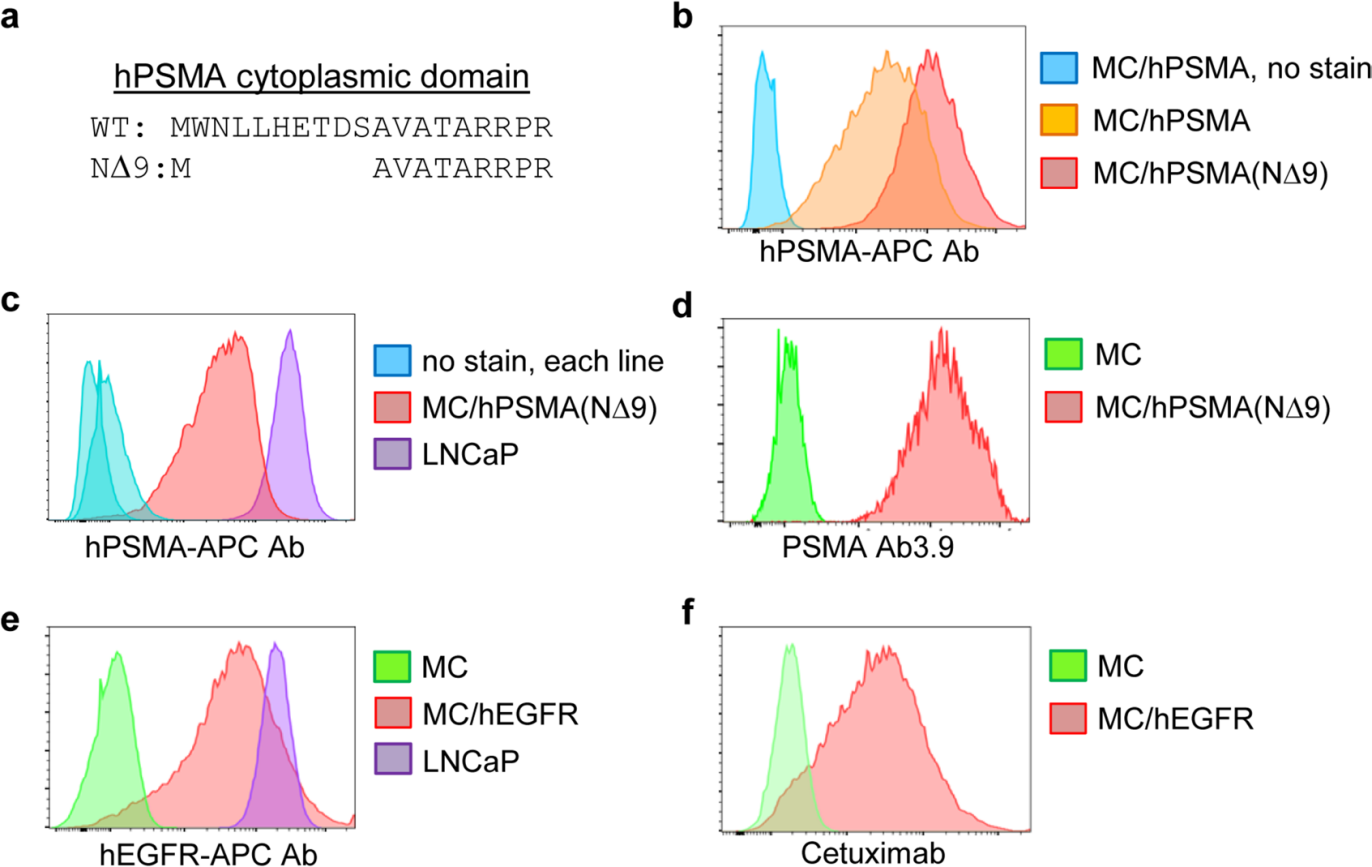
Development of murine prostate cancer lines expressing hPSMA or hEGFR and their detection by PSMA Ab3.9 or Cetuximab. **a** Amino acid sequence of wild-type (WT) hPSMA and its NΔ9 variant lacking the MWNLL internalization domain. **b** Flow cytometry analysis of surface hPSMA on indicated Myc-CaP (MC) lines using commercial hPSMA-APC Ab. **c** Comparison of surface hPSMA expression in human LNCaP cells and MC/hPSMA(NΔ9) cells. **d** Detection of surface hPSMA by PSMA Ab3.9, in conjunction with goat anti-murine IgG-PE secondary Ab, on MC/hPSMA(NΔ9) cells. **e** Detection of surface hEGFR on MC/hEGFR cells and LNCaP cells using hEGFR-Alexa-647 Ab. **f** Detection of hEGFR on MC/hEGFR cells using Cetuximab, with rat anti-human IgG-PE. These data are each from a single assessment

The B6CaP cells we used to demonstrate the efficacy of 5-FU/p50-IMC against murine prostate cancer were obtained from a cancer that developed after FVB/N strain Hi-Myc mice were crossed onto the B6 background [24]. B6CaP cells are maintained as allografts, making it difficult to obtain a uniform malignant population that expresses a transgene that can serve as an antibody or CAR target. We therefore utilized Myc-CaP (MC) cells for our studies, which were developed from FVB/N Hi-Myc mice and are therefore genetically similar to B6CaP cells [25]. MC cells were transduced with retroviral vectors expressing hPSMA or hPSMA(NΔ9), the latter lacking nine cytoplasmic amino acids (Fig. 1a) that includes the MXXXL motif that mediates PSMA internalization [26]. Pooled transductants sorted for high transgene expression were stained with anti-hPSMA-APC Ab, demonstrating ~ threefold higher median cell surface expression of hPSMA(NΔ9) compared with hPSMA (Fig. 1b), although still lower than the endogenous level of hPSMA on the LNCaP human prostate cancer line (Fig. 1c). Surface hPSMA(NΔ9) was also effectively detected by PSMA Ab3.9, which was purified from a hybridoma line and used for our phagocytosis and tumor localization studies (Fig. 1d). We utilized MC/hPSMA(NΔ9) rather than MC/hPSMA cells to more closely model the level of hPSMA expression on human prostate cancer.

MC cells were also transduced with a retroviral vector expressing hEGFR, leading to expression similar to that seen in LNCaP cells (Fig. 1e). hEGFR was effectively detected by both anti-hEGFR-Alexa-647 Ab and Cetuximab, an FDA-approved hEGFR Ab (Fig. 1f).

### PSMA or EGFR antibody increases phagocytosis by p50-deficient macrophages

**Fig. 2:**
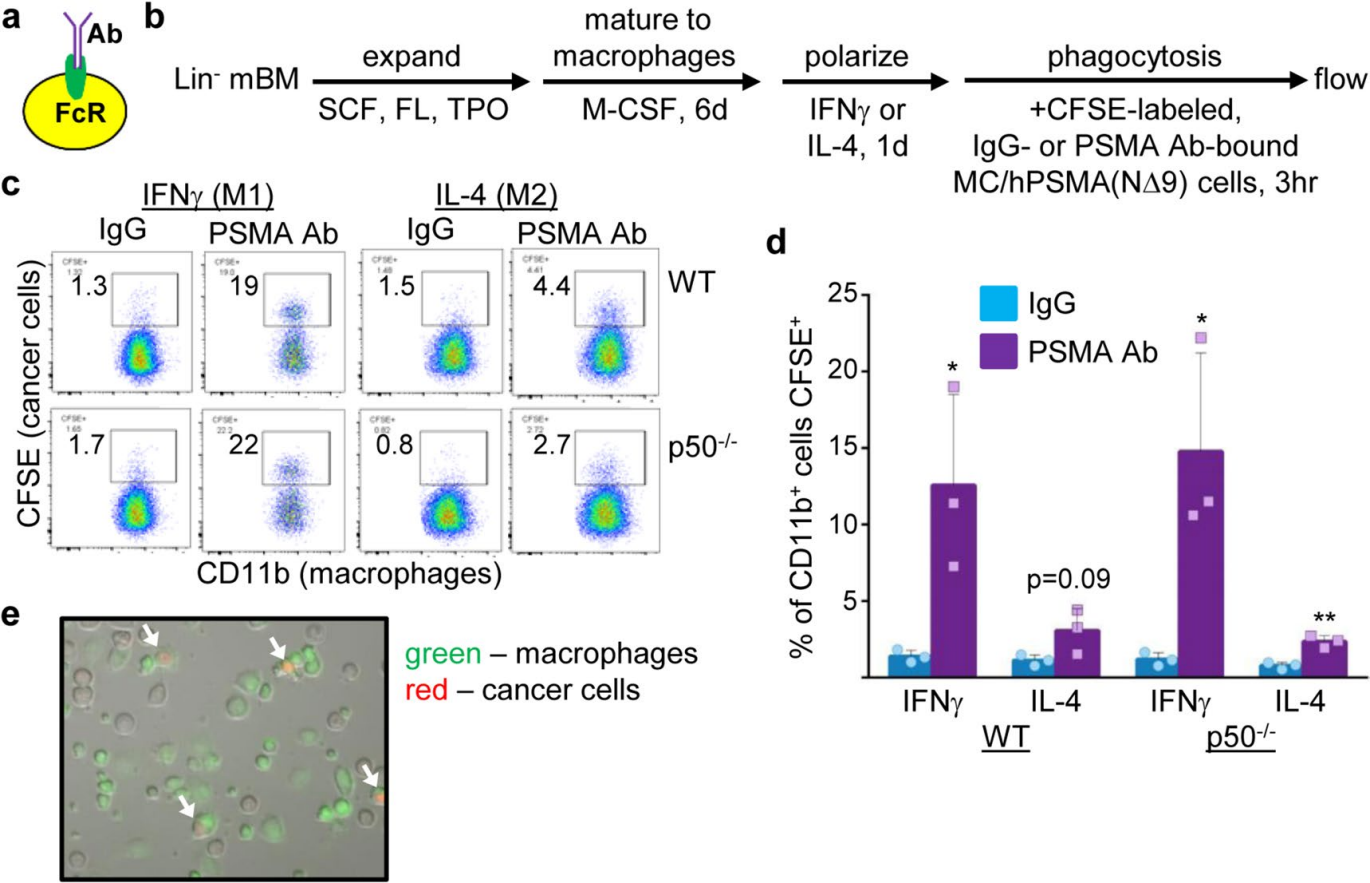
PSMA antibody increases phagocytosis of hPSMA-expressing prostate cancer cells. **a** Diagram of the Fc domain of an Ab bound to a macrophage via the Fc receptor (FcR). **b** Lineage-negative (Lin^−^) WT or p50^−/−^ murine bone marrow (mBM) cells were expanded, differentiated to macrophages using M-CSF, M1- or M2-polarized using IFNγ or IL-4, and co-cultured for 3 h with CFSE-labeled MC/PSMA(NΔ9) that had been incubated with PSMA Ab3.9 or isotype IgG control, as diagrammed. **c** Representative flow cytometry data, previously gating on CD11b^+^ cells. **d** Results of three experiments (one repetition per experiment) evaluating CFSE^+^ cells as a percentage of CD11b^+^ macrophages. (mean, SD) *p < .05, **p < .01. **e** CFSE-labeled macrophages (green) were co-cultured with pHRodo, Red SE-labeled MC/PSMA(NΔ9) cells and PSMA Ab3.9, followed by microscopy (bright field, red, and green channels). Phagocytosed cancer cells are indicated by white arrows

Macrophages can bind the Ab Fc domain via Fc receptors (Fig. 2a). ~ 60% of our immature p50-IMC express surface FcγR2 and FcγR3, and ~ 30% express FcγR4 (Fig. S1a). When differentiated into macrophages, surface expression of FcγR2, FcγR3, and FcγR4 increases to similar levels on both WT and p50^−/−^ macrophages, in IFNγ or IL-4 (Fig. S1b).

We sought to determine whether PSMA Ab increases phagocytosis by p50-IMC-derived macrophages. Lineage-negative WT or p50^−/−^ murine bone marrow cells were expanded, differentiated to adherent macrophages using M-CSF, and then polarized by culture with IFNγ (M1) or IL-4 (M2). Upon release from adherence, macrophages were co-cultured at a 1:2 ratio with CFSE-labeled MC/hPSMA(NΔ9) cells pre-incubated with PSMA Ab3.9 or isotype control. After 3 h of co-culture, all cells were analyzed by flow cytometry for the macrophage marker CD11b and CFSE, as diagramed (Fig. 2b). Macrophages that have phagocytosed MC/hPSMA(NΔ9) cells are CD11b^+^CFSE^+^. Representative flow cytometry data and mean values for the percentage of CD11b^+^ cells that are CFSE^+^ are shown (Fig. 2c, d). On average, PSMA Ab significantly increased phagocytosis, 9- or 12-fold, by M1-polarized WT or p50^−/−^ macrophages, respectively. Although the absolute level of Ab-mediated phagocytosis was ~ sixfold lower by p50^−/−^ macrophages polarized with IL-4 than IFNg, phagocytosis was still significantly increased, by threefold, in the presence of PSMA Ab. Importantly, Ab-mediated phagocytosis by WT and p50^−/−^ macrophages was not significantly different in either culture condition, indicating that the loss of p50 does not impair phagocytic activity of macrophages. To confirm cell internalization, macrophages were labeled with CFSE and co-cultured with PSMA Ab-bound MC/hPSMA(NΔ9) cells labeled with pHrodo, Red SE, which only fluoresces when inside the acidic lysosome. Microscopy verified the presence of red cancer cells inside lysosomes of green macrophages (Fig. 2e). PSMA Ab3.9 did not increase phagocytosis of parental MC cells by WT or p50^−/−^ macrophages in IFNγ or IL-4 (Fig. S2a).

**Fig. 3:**
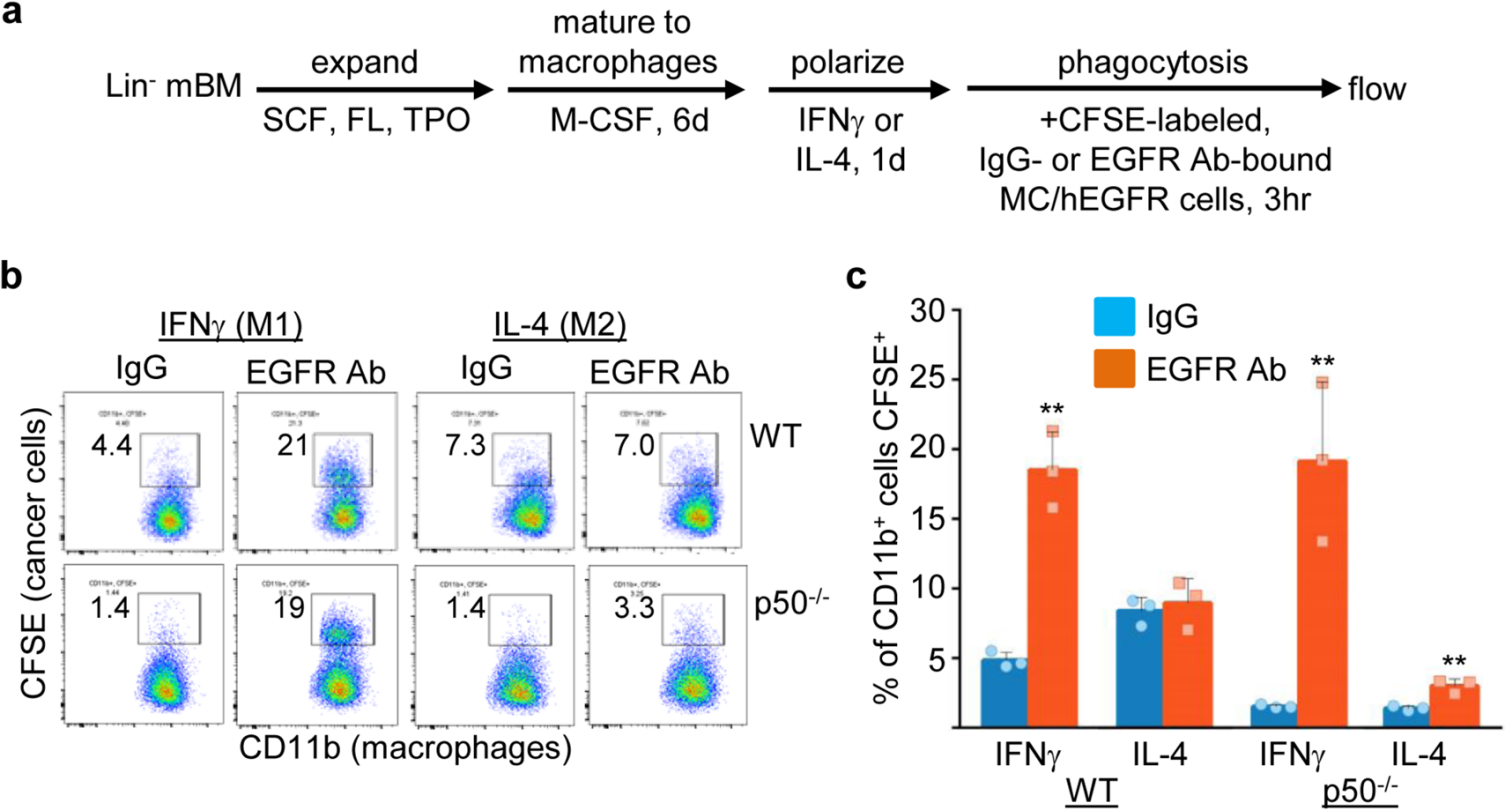
EGFR antibody increases phagocytosis of hEGFR-expressing prostate cancer cells. **a** Lin^−^ WT or p50^−/−^ murine bone marrow (mBM) cells were expanded, differentiated to macrophages using M-CSF, M1- or M2-polarized using IFNγ or IL-4, and co-cultured for 3 h with CFSE-labeled MC/hEGFR cells that had been incubated with EGFR antibody (Cetuximab) or isotype IgG control, as diagrammed. **b** Representative flow cytometry data, previously gating on CD11b^+^ cells. **c** Results of three experiments (one repetition per experiment) evaluating CFSE^+^ cells as a percentage of CD11b^+^ macrophages (mean, SD). *p < .05, **p < .01

WT and p50^−/−^ M1 and M2 macrophages were also co-cultured with CFSE-labeled MC/hEGFR cells that had been incubated with the EGFR Ab Cetuximab or isotype control, as diagrammed (Fig. 3a). Representative flow cytometry data with mean values for the percentage of CD11b^+^ cells that are CFSE^+^ are shown (Fig. 3b, c). EGFR Ab increased phagocytosis 4- or 12-fold by M1-polarized WT or p50^−/−^ macrophages, respectively. EGFR Ab also significantly increased phagocytosis by M2-polarized p50^−/−^ macrophages, by twofold. EGFR Ab did not increase phagocytosis by M2-polarized WT macrophages. Of potential relevance, there was higher baseline phagocytosis by WT, but not p50^−/−^, cells with the human IgG1 isotype control used in the EGFR Ab phagocytosis experiments than with the murine IgG2b isotope control used in the PSMA Ab experiments, perhaps reflecting differential Fc receptor signaling in WT compared with p50^−/−^ macrophages. The absolute level of Ab-mediated phagocytosis was again sixfold lower for M2- compared with M1-polarized p50^−/−^ macrophages, and Ab-mediated phagocytosis by WT versus p50^−/−^ macrophages was not significantly different in IFNγ, but was threefold lower for p50^−/−^ compared to WT macrophages in IL-4 (*p* = 0.003). EGFR Ab did not increase phagocytosis of parental MC cells (Fig. S2b).

### PSMA.CAR10.3 increases phagocytosis by p50-deficient macrophages

**Fig. 4:**
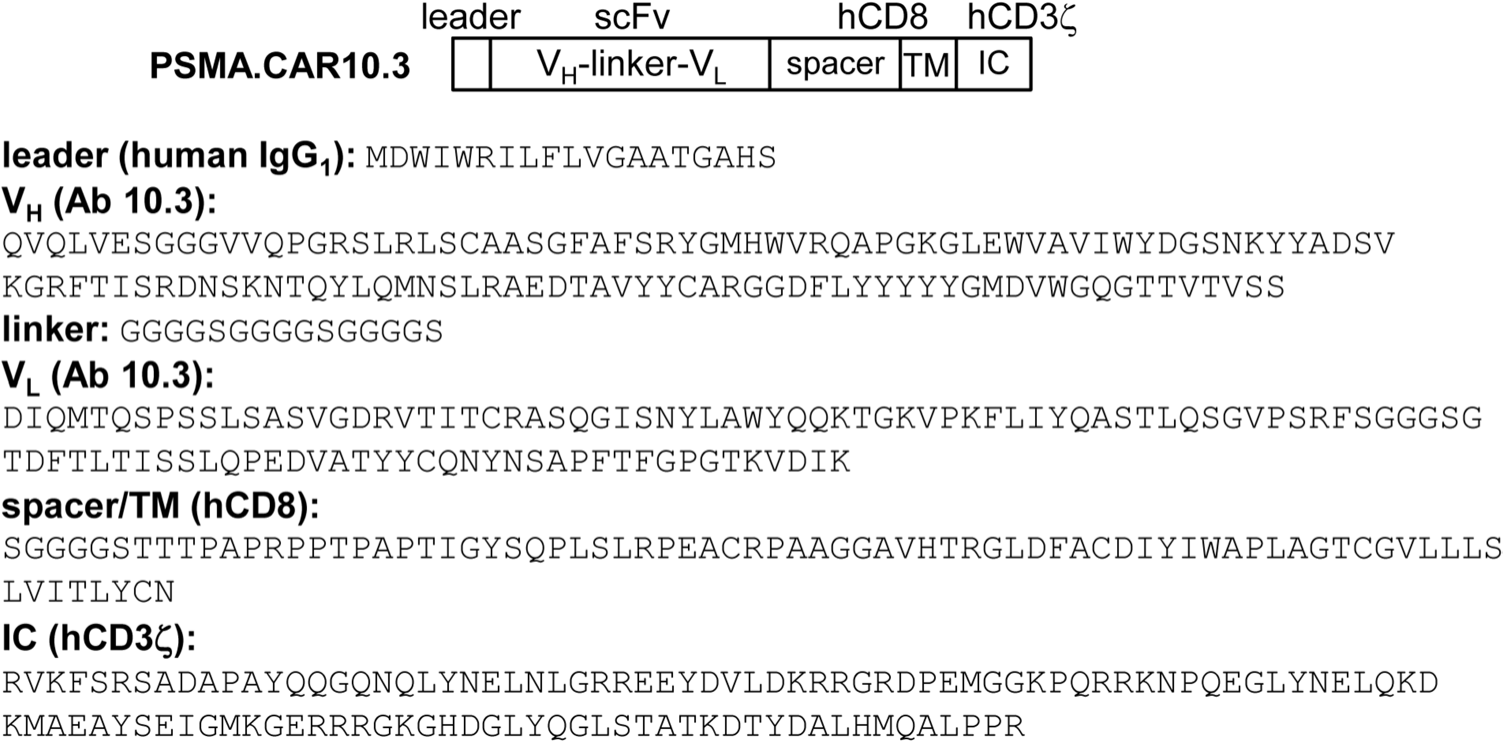
Development of a fully humanized PSMA.CAR10.3. Diagram of PSMA.CAR10.3, containing a leader sequence from human IgG1, an scFv domain derived from PSMA Ab10.3 by connecting its V_H_ and V_L_ domains with a linker peptide, spacer and trans-membrane (TM) domains from human CD8 (hCD8), and the intracellular (IC) signaling domain from human CD3ζ (hCD3ζ). PSMA Ab10.3 was developed from mice harboring only human immunoglobulin genes. The amino acid sequences of the PSMA.CAR10.3 domains are also shown

As the PSMA Ab3.9 DNA sequence is not available, we utilized the sequence of PSMA Ab10.3 to design a PSMA.CAR [19]. PSMA.CAR10.3 was assembled by combining an IgG1 leader peptide, V_H_ and V_L_ domains from PSMA Ab10.3, a (GGGGS)_3_ linker peptide connecting the variable domains, spacer and trans-membrane domains from human CD8, and an intracellular signaling domain from human CD3ζ (Fig. 4). Of note, the hCD3ζ domain is sufficient for mediating phagocytosis by human or murine macrophages [27, 28], and PSMA.CAR10.3 is fully humanized.

**Fig. 5:**
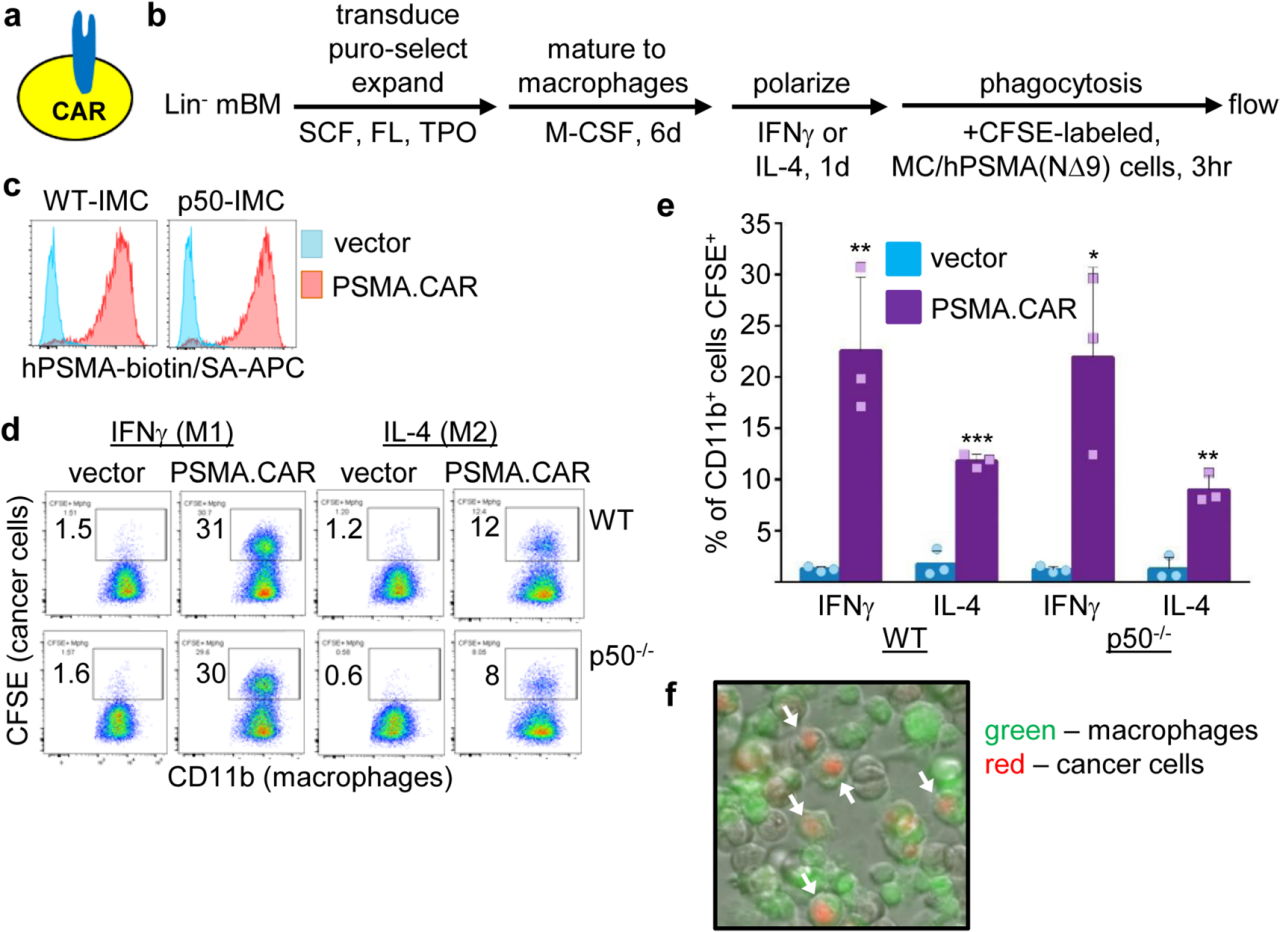
PSMA.CAR10.3 increases phagocytosis of hPSMA-expressing prostate cancer cells. **a** Diagram of a CAR expressed on a macrophage. **b** WT or p50^−/−^ Lin^−^ murine bone marrow cells were expanded and transduced with vector or PSMA.CAR10.3, differentiated to macrophages, M1- or M2-polarized, and co-cultured with CFSE-labeled MC/PSMA(NΔ9) cells, as diagrammed. **c** Flow cytometry of vector- and PSMA.CAR10.3-transduced IMC, after puromycin-selection, using hPSMA-biotin and SA-APC. **d** Representative flow cytometry showing phagocytosis by vector versus CAR-expressing macrophages, previously gating on CD11b^+^ cells. **e** Results of three experiments (one repetition per experiment) evaluating CFSE^+^ cells as a percentage of CD11b^+^ macrophages (mean, SD). *p < .05, **p < .01, ***p < .001. **f** CFSE-labeled macrophages expressing PSMA.CAR10.3 (green) were combined with pHRodo, Red SE-labeled MC/PSMA(NΔ9) cells, followed by microscopy (bright field, red, and green channels). Phagocytosed cancer cells are indicated by white arrows

A diagram of a macrophage with a surface CAR is shown (Fig. 5a). To evaluate the impact of PSMA.CAR10.3 expression on phagocytosis, lineage-negative WT or p50^−/−^ murine bone marrow cells were expanded, transduced with MIPuro (vector) or MIPuro-PSMA.CAR10.3, differentiated into adherent macrophages using M-CSF, and M1- or M2-polarized. After release from adherence, macrophages were co-cultured with CFSE-labeled MC/hPSMA(NΔ9) cells, followed by flow cytometry for CD11b and CFSE, as diagramed (Fig. 5b). Uniform, high-level expression of PSMA.CAR10.3 in WT and p50^−/−^ IMC, as well as the ability of this CAR to interact with hPSMA, was verified by flow cytometry using hPSMA-biotin and streptavidin-APC (Fig. 5c). Representative flow cytometry data and mean values for the percentage of CD11b^+^ macrophages that are CFSE^+^ are shown (Fig. 5d, e). On average, PSMA.CAR10.3 increased phagocytosis 18-fold by M1-polarized WT and p50^−/−^ macrophages and sevenfold by M2-polarized WT and p50^−/−^ macrophages. The absolute level of CAR-mediated phagocytosis was 2.4-fold lower for M2- compared with M1-polarized p50^−/−^ macrophages. CAR-mediated phagocytosis by p50^−/−^ macrophages was similar to that of WT macrophages in IFNγ, but was mildly reduced compared to WT in IL-4 (*p* = 0.035). Interestingly, when polarized with IL-4, a component of the immune-suppressive tumor environment, p50^−/−^ macrophage phagocytosis mediated by PSMA.CAR10.3 was ~ fourfold greater than that of PSMA Ab3.9. To verify phagocytosis, CFSE-labeled M1 macrophages expressing PSMA.CAR10.3 were co-cultured with MC/hPSMA(NΔ9) cells labeled with pHrodo Red SE, followed by microscopy, which again confirmed that cancer cells were engulfed by macrophages (Fig. 5f). PSMA.CAR10.3 did not increase phagocytosis of parental MC cells by WT or p50^−/−^ macrophage in IFNγ or IL-4 (Fig. S2c).

### PSMA or EGFR antibody increases localization of p50-IMC to prostate cancer tumors

Tumor localization studies were conducted using MC lines expressing human rather than murine PSMA or EGFR due to the specificity of our Abs and CAR. MC cells stably transfected with murine PSMA (mPSMA) express abundant transgene, as assessed by Western blot analysis (Fig. S3a). Flow cytometry using a non-cleavable PSMA enzymatic active site ligand linked to a generation 4 (G4) polyamidoamine dendrimer nanoparticle and Cy5 [29] readily detected mPSMA on MC/mPSMA cells (Fig. S3b, left). PSMA Ab3.9, however, did not detect surface mPSMA in flow cytometry (Fig. S3b, right). Similarly, Cetuximab binds hEGFR but not mEGFR [30]. Additionally, M1-polarized p50^−/−^ macrophages expressing PSMA.CAR10.3 effectively phagocytosed MC/hPSMA(NΔ9) but not MC/mPSMA cells (Fig. S3c), indicating that PSMA.CAR10.3 also binds human but not murine PSMA.

Parental MC cells readily produced tumors in syngeneic FVB/N mice. MC/hPSMA cells, however, only rarely formed tumors in FVB/N mice but readily formed tumors in immune-deficient NSG mice (Fig. S4a). The few MC/hPSMA tumors that arose late in FVB/N mice had largely lost surface hPSMA, whereas MC/hPSMA tumors retained hPSMA in NSG mice; in addition, MC/mPSMA cells readily form tumors in FVB/N mice that retain surface mPSMA (Fig. S4b). Similarly, TRAMP-C1/hPSMA cells generated tumors in NSG but not in syngeneic C57BL/6 mice (Fig. S4c, d). These results indicate that hPSMA is not tolerated by immune-competent mice. Similarly, we find that MC cells expressing hEGFR do not retain hEGFR in syngeneic mice, whereas MC/hEGFR cells express abundant hEGFR in NSG mice (Fig. S5).

In an effort to render FVB/N mice tolerant to hPSMA, we developed transgenic AR_2_Pbn-hPSMA mice. Although progeny of two founders express hPSMA RNA in their prostates, neither murine nor human PSMA protein was detected (Fig. S6), and MC/hPSMA cells did not form tumors in these mice. Murine PSMA protein was detected in the prostate of a Hi-Myc mouse (Fig. S6), which develop prostate cancer, as described previously [31]. Because we lacked an immune-competent host that tolerates hPSMA or hEGFR, we assessed the ability of hPSMA Ab, hEGFR Ab, or PSMA.CAR10.3 to increase p50-IMC localization to tumors expressing hPSMA(NΔ9) or hEGFR in NSG mice. Use of an entirely murine system (p50-IMC, MC lines, NSG mice) ensures that species-specific extra-cellular matrix:cell interactions do not complicate data interpretation.

**Fig. 6:**
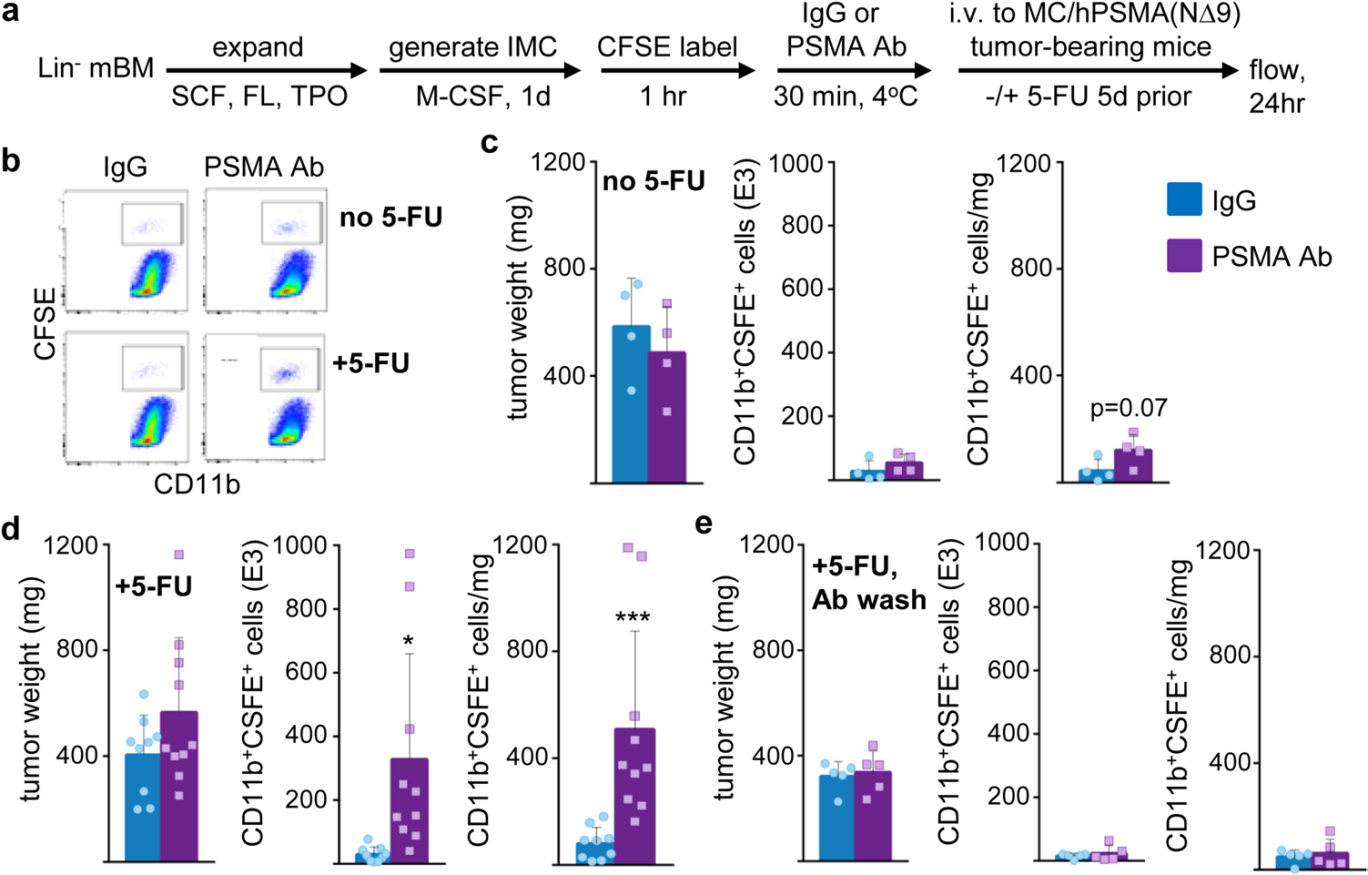
PSMA antibody increases p50-IMC localization to hPSMA-expressing prostate cancer tumors when given after 5-FU. **a** Lin^−^ p50^−/−^ murine bone marrow cells were expanded, cultured with M-CSF for one day to obtain p50-IMC, CFSE-labeled, incubated with 100 μg PSMA antibody or isotype IgG control for one hour on ice, and injected into NSG mice bearing subcutaneous tumors derived from MC/CaP-hPSMA(NΔ9) cells in Matrigel, with or without 5-FU (112.5 mg/kg i.p.) given to the mice five days prior to cell injection, followed by tumor flow cytometry at 24 h, as diagrammed. **b** Representative CFSE/CD11b flow cytometry. **c** Tumor weight, total tumor CD11b^+^CSFE^+^ cells, and CD11b^+^CSFE^+^ cells per mg of tumor in mice that did not receive 5-FU (mean, SE; *n* = 4 per group, from one experiment). **d** Tumor weight, total tumor CD11b^+^CSFE^+^ cells, and CD11b^+^CSFE^+^ cells per mg of tumor in mice that did receive 5-FU (mean, SD; IgG *n* = 9 and PSMA Ab *n* = 10; data are combined from two experiments). **e** The experiment in d was repeated with removal of excess PSMA Ab or isotype control by centrifugation, supernatant aspiration, and resuspension in HBSS prior to injection (*n* = 5 per group, from one experiment). *p < 0.05, **p < 0.01

**Fig. 7:**
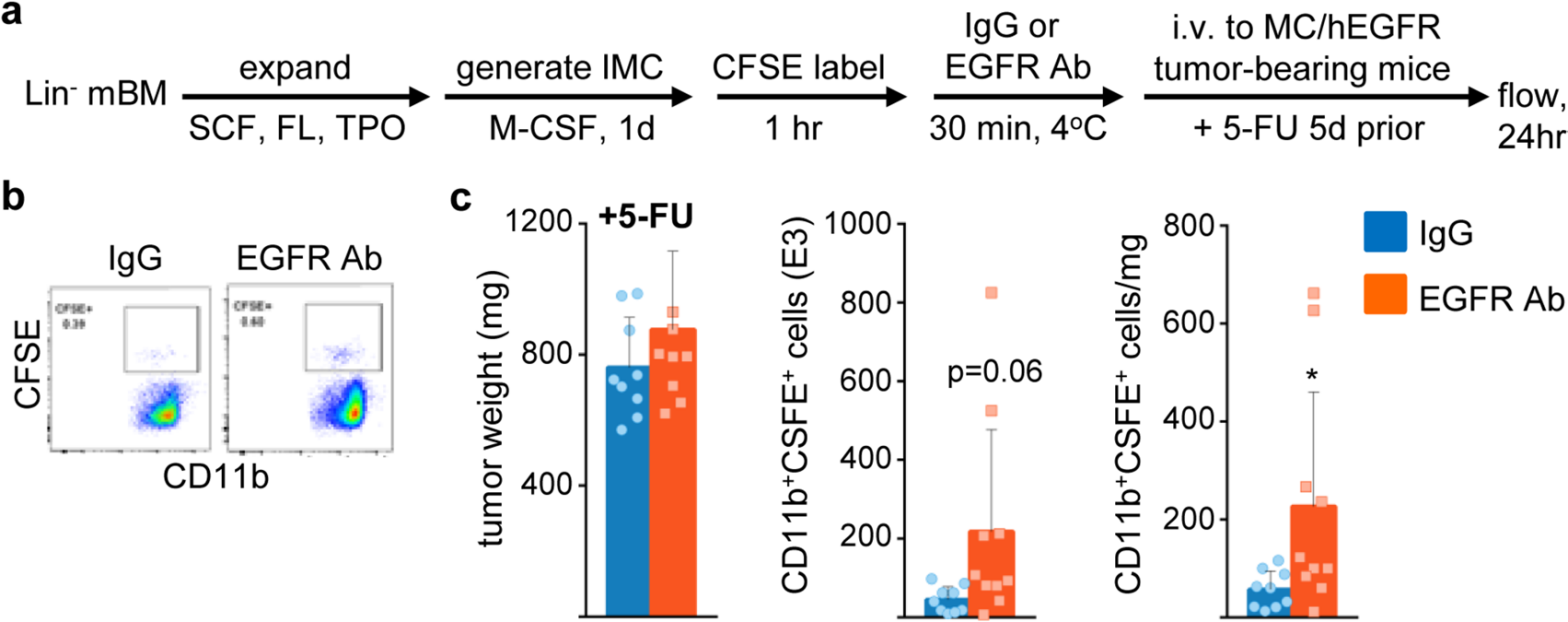
EGFR antibody increases p50-IMC localization to hEGFR-expressing prostate cancer tumors. **a** Lin^−^ p50^−/−^ murine bone marrow cells were expanded, cultured with M-CSF for one day, CFSE-labeled, incubated with 100 μg EGFR antibody or IgG control, and injected into NSG mice bearing subcutaneous tumors derived from MC/hEGFR cells, followed by tumor flow cytometry at 24 h, as diagrammed. Mice received 5-FU five days prior to p50-IMC injection. **b** Representative CFSE/CD11b flow cytometry. **c** Tumor weight, total tumor CD11b^+^CFSE^+^ cells, and CD11b^+^CFSE^+^ cells per mg of tumor (mean, SD; IgG *n* = 9 and EGFR Ab *n* = 8; data are combined from two experiments)

CFSE-labeled p50-IMC were combined with PSMA Ab3.9 or isotype control and injected intravenously into NSG mice bearing MC/hPSMA(NΔ9) tumors, as diagramed (Fig. 6a). Tumors were isolated 24 h later and subjected to CD11b/CFSE flow cytometry, with representative data shown (Fig. 6b). PSMA Ab3.9 did not increase tumor localization in our initial experiment (Fig. 6c). We next evaluated tumor localization after administration of 5-FU prior to p50-IMC injection and observed that PSMA Ab3.9 then significantly increased the total number of tumor-associated p50-IMC progeny as well as the number of IMC per mg of tumor weight (Fig. 6d). In these experiments, PSMA Ab3.9 was in great excess above what was bound to p50-IMC via Fc receptors. When we washed out excess Ab prior to intravenous administration, PSMA Ab3.9 did not increase p50-IMC tumor localization (Fig. 6e). Similarly, combining hEGFR Ab (Cetuximab) with p50-IMC increased localization of p50-IMC to MC/hEGFR tumors in NSG mice, when administered after a dose of 5-FU, with total IMC per mg of tumor reaching significance (Fig. 7).

### PSMA.CAR 10.3 increases localization of p50-IMC to prostate cancer tumors

**Fig. 8:**
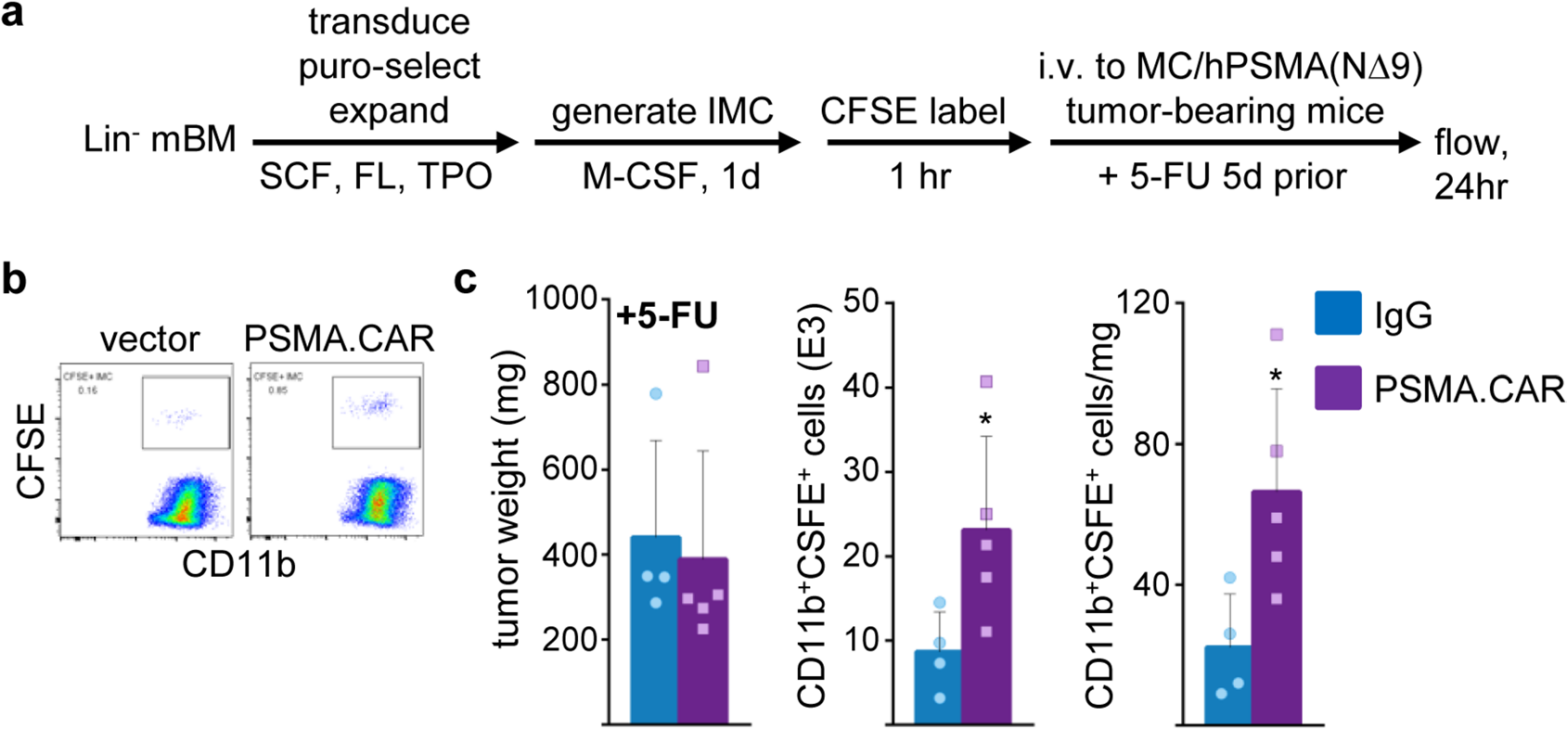
PSMA.CAR10.3 increases p50-IMC localization to hPSMA-expressing prostate cancer tumors. **a** Lin^−^ p50^−/−^ murine bone marrow cells were expanded, transduced with vector or PSMA.CAR10.3, cultured with M-CSF, CFSE-labeled, and injected into NSG mice bearing tumors derived from MC/CaP-hPSMA(NΔ9) cells, and analyzed by flow cytometry 24 h later, as diagrammed. Mice received 5-FU five days prior to p50-IMC injection. **b** Tumor weight, total tumor CD11b^+^CFSE^+^ cells, and CD11b^+^CFSE^+^ cells per mg of tumor (mean, SD; *n* = 4 for vector and *n* = 5 for PSMA.CAR10.3, from one experiment)

Lineage-negative p50^−/−^ marrow cells were transduced with vector or PSMA.CAR10.3, cultured in M-CSF to generate p50-IMC, CFSE-labeled, and injected five days after a dose of 5-FU into NSG mice bearing MC/hPSMA(NΔ9) tumors. After 24 h, tumors were isolated and analyzed by flow cytometry, as diagramed (Fig. 8a). Representative flow plots are shown (Fig. 8b). PSMA.CAR10.3 significantly increased p50-IMC localization to prostate cancer tumors expressing hPSMA(NΔ9) (Fig. 8c). Of note, CAR expression only minimally affects expression of myeloid-lineage makers on p50-IMC (Fig. S7).

## Discussion

p50-IMC macrophage and dendritic cell progeny may activate T cells by secreting pro-inflammatory cytokines as well as by phagocytosing cancer cells and cross-presenting neoantigens to CD8^+^ T cells. We find that the absence of p50 does not compromise the ability of p50^−/−^ macrophages to phagocytose MC prostate cancer lines and that addition of PSMA or EGFR Ab or the expression of PSMA.CAR10.3 augments phagocytosis by p50-deficient macrophages in both IFNγ and IL-4, the latter mimicking the immune-suppressive tumor microenvironment. Additionally, we find that PSMA.CAR10.3 is more effective at increasing phagocytosis than PSMA Ab in IL-4. PSMA Ab and PSMA.CAR10.3 also increased localization of p50-IMC to MC tumors expressing hPSMA, and EGFR Ab increased localization of p50-IMC to MC tumors expressing hEGFR. These findings suggest that the addition of antibodies or CARs targeting PSMA or EGFR may augment the ability of p50-IMC to induce a T cell response and increase efficacy against prostate cancer in patients not only by increasing the numbers of p50-IMC in the tumors, but also by enhancing phagocytosis of tumor cells.

Our results demonstrate the necessity of a preceding 5-FU dose to augment PSMA Ab-mediated p50-IMC tumor localization; this phenomenon may also account for the requirement for 5-FU to enable p50-IMC efficacy against B6CaP tumors [8]. Potentiation of p50-IMC tumor localization by 5-FU likely reflects both suppression of bone marrow production of competing blood monocytes and ~ twofold reduction of tumor macrophages [32, 33]. The addition of Ab provides a potentially practical means to augment p50-IMC efficacy. We find that removal of excess Ab prevents augmentation of tumor localization, guiding the optimal approach to combining p50-IMC with Ab. On the other hand, our data suggest that PSMA.CAR10.3 may more effectively augment phagocytosis and thereby MHC-mediated T cell activation in the tumor microenvironment. PSMA.CAR10.3 is a fully humanized CAR, which could prove useful for clinical application, avoiding the development of human anti-murine Abs that can occur when CARs containing murine components are utilized. Of note, tumor cell clearance via increased phagocytosis, as seen with Ab or CAR in vitro, is not likely the predominant mechanism of p50-IMC efficacy in vivo, given the small numbers of p50-IMC that reach tumor (~ 2% of tumor myeloid cells in the B6CaP model) and the dependence of anti-tumor efficacy on T cell activation [8].

We found a lack of tolerance for hPSMA in both FVB/N and B6 mice and a lack of tolerance for hEGFR in FVB/N mice. RM-1 prostate cancer cells expressing human PSMA form tumors in syngeneic B6 mice; however, tumor regression occurs after 14 days in a subset of mice, and subsequent challenge with hPSMA-expressing but not control cells is rejected, suggesting induction of anti-hPSMA immunity. In addition, retention of high-level surface hPSMA expression by tumors has not been documented in this or the related RM-1/PGLS model, with tumor interaction with radio-labeled PSMA ligand potentially reflecting low-level surface hPSMA [34].

Our inability to express hPSMA in the mouse prostate may reflect the inability of the normal prostate to tolerate high levels of PSMA, in contrast to prostate cancer, a phenomenon worthy of further study to potentially reveal a novel vulnerability in prostate cancer. We utilized the AR_2_-Pbn regulator elements with the intent of both obtaining hPSMA-tolerant mice and breeding AR_2_-Pbn-hPSMA mice with AR_2_-Pbn-c-Myc (Hi-Myc) mice, the latter to determine the effect of increased PSMA on Hi-Myc prostate cancer progression and to develop a model of endogenous murine prostate cancer that we could target with hPSMA-directed p50-IMC. In the future, we could express hPSMA from the mPSMA genomic locus, which has been shown to enable hPSMA tolerance [35].

Ab- and CAR-mediated tumor localization studies were focused on p50-IMC rather than WT-IMC due to their proven enhanced efficacy and clinical relevance. We plan to evaluate and compare WT-IMC once we have an immune-competent model tolerant to hPSMA or hEGFR, so that our results can be correlated with anti-tumor efficacy. Once we establish an immune-competent model, it will also be of interest to use histologic or spatial transcriptomics methods to determine the location of p50-IMC and their myeloid progeny within tumors in relation to other immune cells, such as T cells and B cells, potentially within tertiary lymphoid structures such as those that predict and arise in response to checkpoint inhibition [36, 37].

p50-IMC offer a means to activate global endogenous T cell immunity against multiple neoantigens, with efficacy potentially increased by the addition of PSMA or EGFR Abs or CARs, and by T cell checkpoint inhibition to overcome subsequent T cell exhaustion. To facilitate clinical translation, we have optimized highly efficient CRISPR/Cas9 gene-editing of the *NFKB1* alleles encoding NF-κB p50 in human marrow CD34^+^ hematopoietic stem/progenitor cells, followed by their robust expansion and conversion to p50-IMC (T. Barberi and A. Friedman, unpublished).

## Supplementary Information

Below is the link to the electronic supplementary material.

Supplementary file1 (PDF 746 KB)

## Data Availability

Data is provided within the manuscript or supplementary information files.

## Abbreviations

5-FU: 5-Fluorouracil
AA: Antibiotic/antimycotic
Ab: Antibody
ADT: Androgen-deprivation therapy
AR: Androgen receptor
CAR: Chimeric antigen receptor
CFSE: Carboxyfluorescein succinimidyl ester
EGFR: Epidermal growth factor receptor
FL: Flt3 ligand
IFNγ: Interferon γ
IL-4: Interleukin-4
IMC: Immature myeloid cells
MC: Myc-CaP cells
mCRPC: Metatastic, castration-resistant prostate cancer
M-CSF: Monocyte colony-stimulating factor
NSG: NOD scid γ mice
p50: NF-κB p50
Pbn: Probasin
PSMA: Prostate-specific membrane antigen
SCF: Stem cell factor
SD: Standard deviation
TPO: Thrombopoietin
ULA: Ultra-low attachment
WT: Wild-type
