# Supplementary material for: PSMA antibody, humanized PSMA.CAR10.3, or Cetuximab increases prostate cancer localization of NF-κB p50-deficient immature myeloid cells (p50-IMC) and phagocytosis by their macrophage progeny
Source: Cancer Immunol Immunother. 2025 Feb 4;74(3):95. doi: 10.1007/s00262-024-03939-4 (PMC11794921; doi:10.1007/s00262-024-03939-4)
Supplement: Supplementary file 1 — Supplementary file1 (PDF 746 KB) [file 262_2024_3939_MOESM1_ESM.pdf]

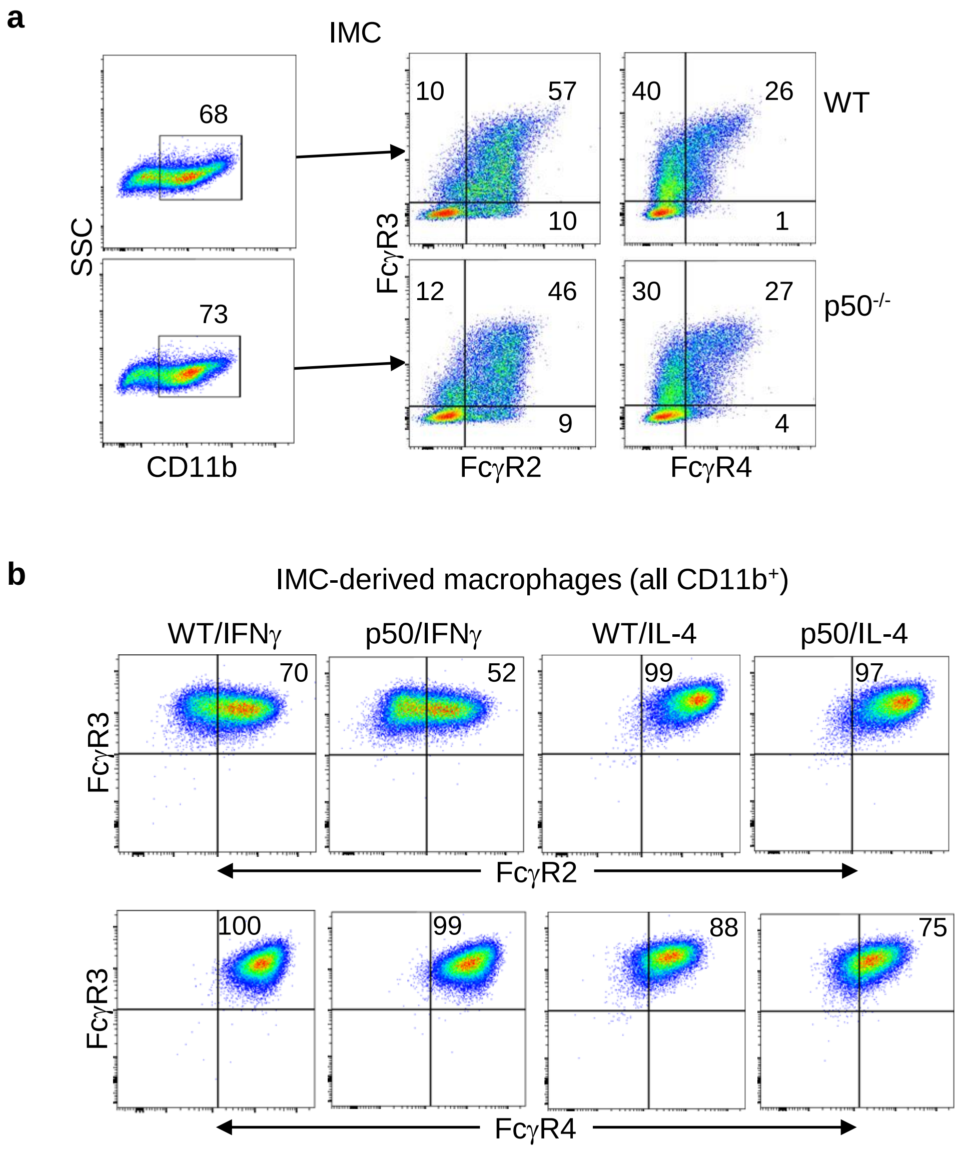

Fig. S1 Fc Receptor expression on IMC and IMC-derived macrophages. **a** WT and p50$^{-/-}$ murine bone marrow cells were expanded for seven days, transferred to M-CSF for 24 hours to generate IMC, and assessed for surface FcγR2, FcγR3, and FcγR4 expression by flow cytometry, gating on CD11b^{+} cells. **b** WT-IMC and p50-IMC were differentiated to macrophages using M-CSF for six days, M1 or M2 polarized using IFNγ or IL-4 for 24 hours, and evaluated similarly. These IMC and macrophage Fc receptor expression data are from a single experiment.

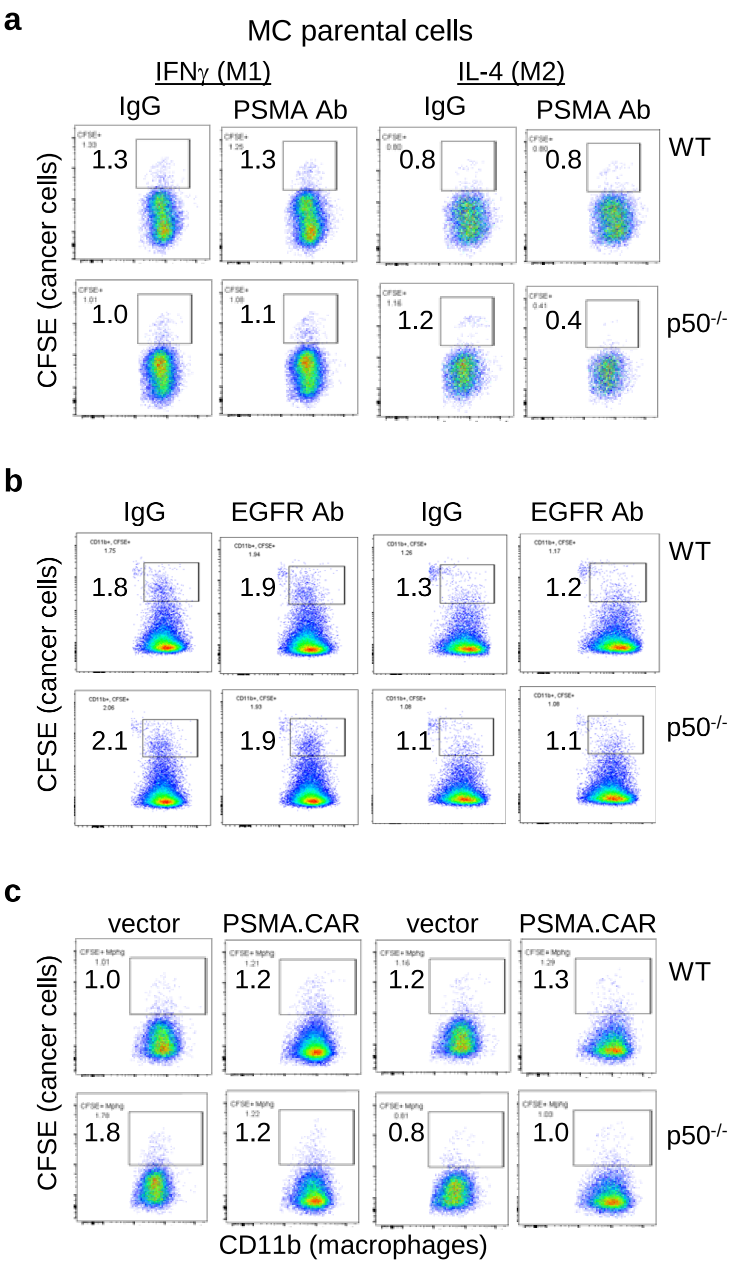

Fig. S2 Phagocytosis assays with parental MC cells. **a, b** Lineage-negative WT or p50$^{-/-}$ murine bone marrow cells were expanded, differentiated to macrophages using M-CSF, M1 or M2 polarized using IFNγ or IL-4, and co-cultured for 3 hours with CFSE-labeled MC parental cells that had been incubated with PSMA Ab3.9, EGFR Ab (Cetuximab), or isotype IgG control, and subjected to flow cytometry for CFSE and CD11b. **c** WT or p50$^{-/-}$ murine bone marrow cells were expanded and transduced with vector or PSMA.CAR10.3, differentiated to macrophages, M1 or M2 polarized, mixed with CFSE-labeled MC parental cells, and subjected to flow cytometry for CFSE and CD11b. Data shown are after gating on CD11b^{+} cells. These phagocytosis assays with parental MC cells are each from a single experiment.

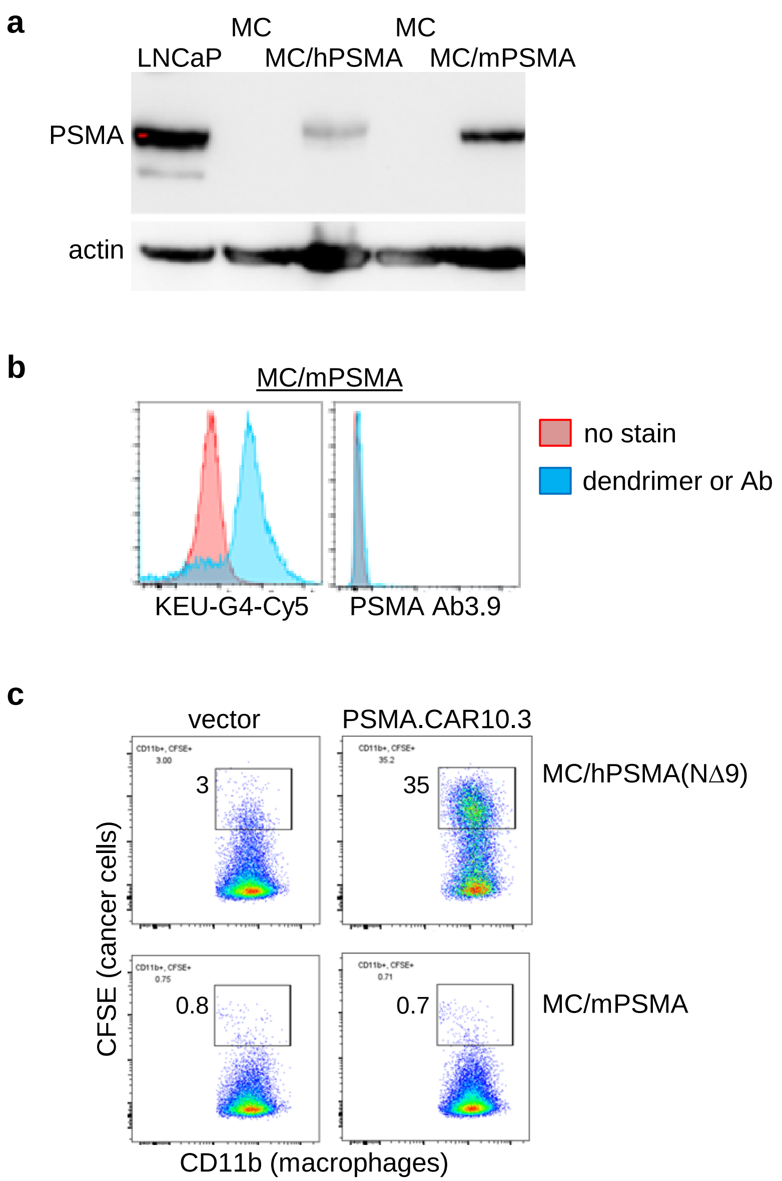

Fig. S3 Murine PSMA does not interact with PSMA Ab3.9 or PSMACAR10.3. **a** Western blot of LNCaP, MC, MC/hPSMA, and MC/mPSMA cells for PSMA, using anti-murine/human PSMA Ab, or β-actin. **b** MC cells expressing murine PSMA (mPSMA) were subjected to flow cytometry with either KEU-G4-Cy5 PAMAM dendrimer (left) or PSMA Ab3.9 and goat anti-murine PE (right). KEU (lysine and glutamic acid connected by a non-hydrolysable urea linkage) binds the murine or human PSMA enzymatic active site. **c** $p50^{-/-}$ lineage-negative murine bone marrow cells were expanded and transduced with MIPuro vector or MIPuro-PSMA.CAR10.3, differentiated to macrophages, M1 polarized, and co-cultured for 3 hours with CFSE-labeled MC/hPSMA(NΔ9) or MC/mPSMA cells, followed by flow cytometry for CD11b and CFSE. The percentage of $CD11b^{+}$ macrophages that were $CFSE^{+}$ are indicated for each MC line. These data are each from a single experiment.

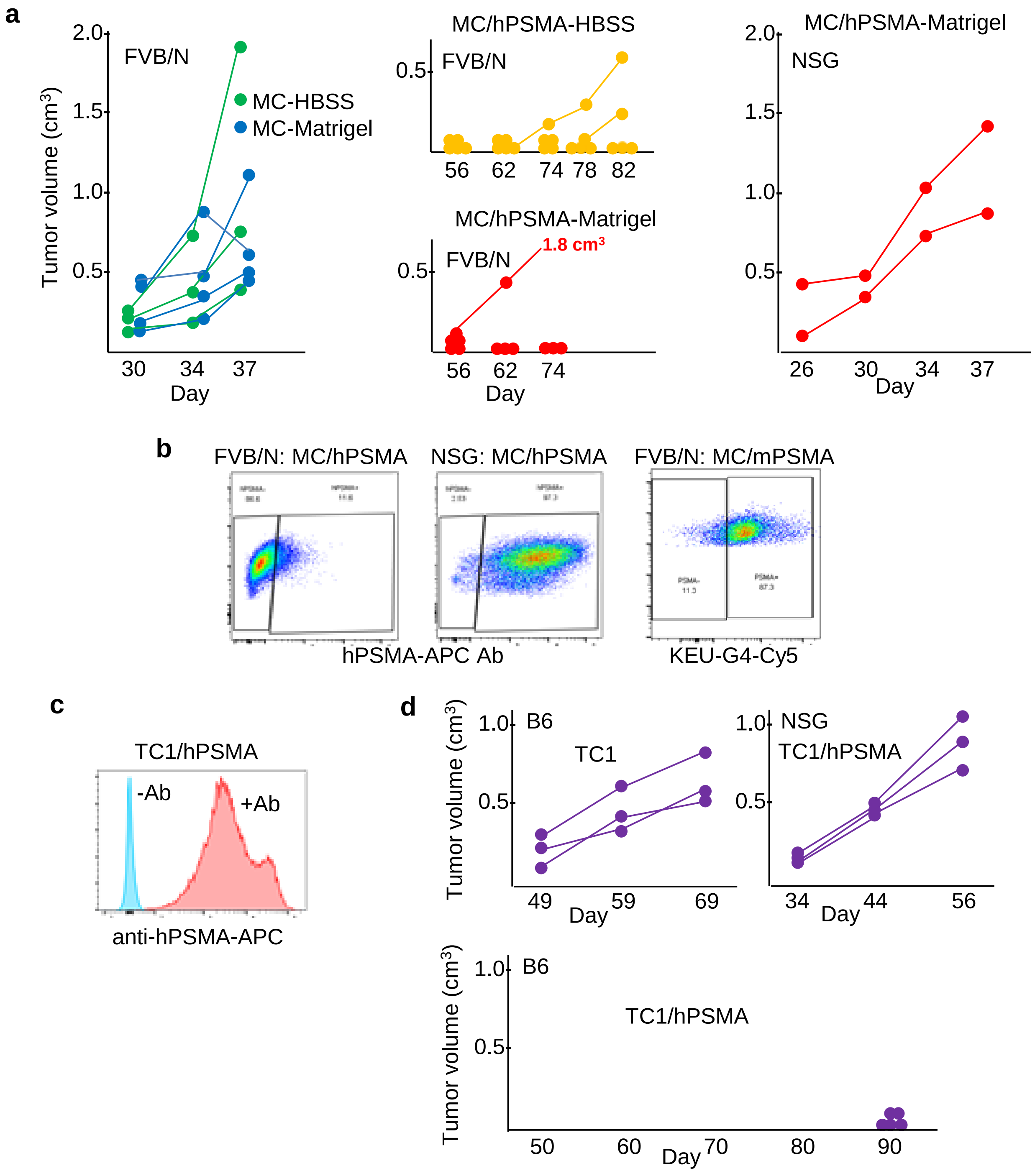

Figure S4. Human PSMA is not tolerated by immune-competent, syngeneic mice. **a** MC or MC/hPSMA tumor growth in FVB/N mice in HBSS or Matrigel and in NSG mice in Matrigel. Each line represents a single tumor. MC/hPSMA dots plotted just above the x-axis (two rows deep on some days) represent mice in which no tumors formed after the indicated number of days (n=18 for HBSS, n=9 for Matrigel). One MC/hPSMA tumor reached 1.8 cm^3 on day 74. **b** Surface PSMA expression on MC/hPSMA tumors in FVB/N or NSG mice, detected with hPSMA-APC Ab, and on a MC/mPSMA tumor in FVB/N mice, detected with the KEU PSMA active site ligand linked to a PAMAM dendrimer and Cy5. **c** Surface hPSMA on TRAMP-C1 (TC1) cells was detected by flow cytometry. **d** TC1 tumor growth in C57BL/6 (B6) mice (n=3), TC1/hPSMA tumor growth in NSG mice (n=3), and lack of TC1/hPSMA tumor growth in B6 mice (n=5) at day 90. Each mouse was inoculated subcutaneously on day 0 with 5E6 TC1 cells in HBSS.

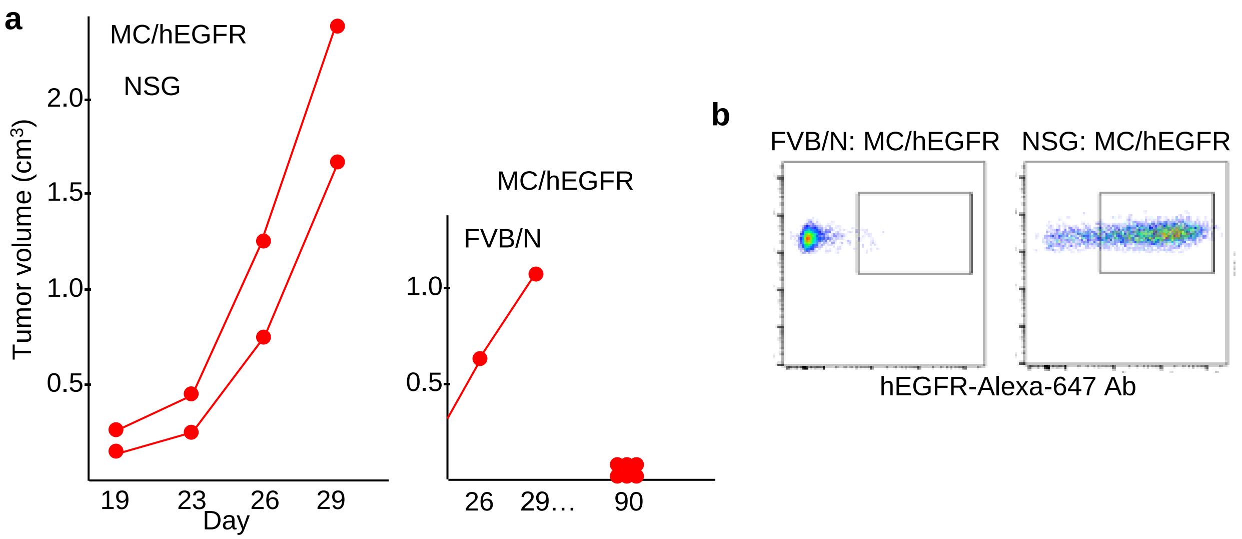

Fig. S5. Human EGFR is not tolerated by immune-competent, syngeneic mice. **a** MC/hEGFR tumor growth in NSG (n=2) and FVB/N (n=7) mice in HBSS. **b** Human EGFR surface expression on MC/hEGFR tumors in an FVB/N and an NSG mouse was evaluated by flow cytometry.

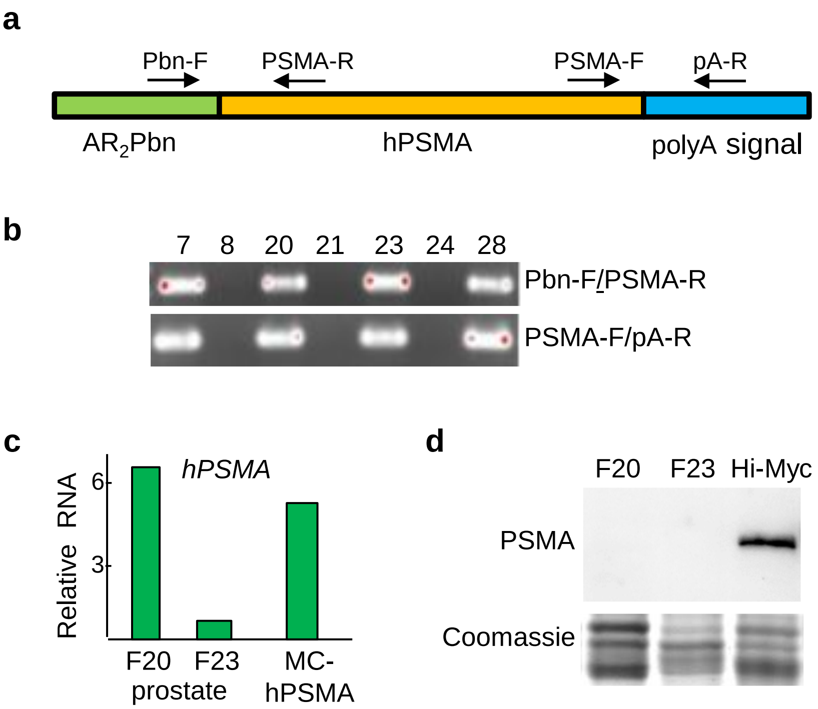

Fig. S6 AR_2Pbn-hPMSA transgenic mice express hPSMA RNA but not protein in the prostate. **a** Diagram of the AR_2Pbn-hPMSA transgene and PCR primer pairs. AR - androgen receptor; Pbn - probasin promoter. polyA - SV0 splice and polyadenylation site. **b** Detection of four transgenic FVB/N founders by tail clip DNA PCR. Data for seven of 34 mice screened are shown. **c** Total RNA from founders 20 and 23 (F20, F23) prostates and from MC/hPSMA cells were analyzed by qRT-PCR for *hPSMA*, relative to ribosomal protein *S16* mRNA. Female (3 of 11) but no male (0 of 17) offspring of founder 7 carried the transgene; no offspring from founder 28 carried the transgene. **d** Western blot for PSMA in the ventral lobes of prostates from F20 and F23 transgenic mice and from a Hi-Myc mouse, using anti-murine/human PSMA Ab, with Coomassie stain as loading control. Proteins were prepared by homogenization of snap frozen prostate lobes after addition of RIPA buffer with protease inhibitors.

Primers for tail PCR were:

Pbn-F: 5'-GACACTGCCCATGCCAATC, PSMA-R: 5'-GATGTGTTGAAAATCTCATTTCC,
PSMA-F: 5'-CTTTGACAAAAGCAACCCAATAG, and pA-R: 5'-TCAGTTCCATAGGTTGGAATC.

Primers for RNA analysis were:

hPSMA-F: 5'-CTGAAAAAGGAAGGGTGGAGACC, hPSMA-R: 5'-GTCTTGAATTCTCCTCTGCCCA,
mS16-F: 5'-CTTGGAGGCTTCATCCACAT, and mS16-R: 5'-ATATTCGGGTCCGTGTGAAG.

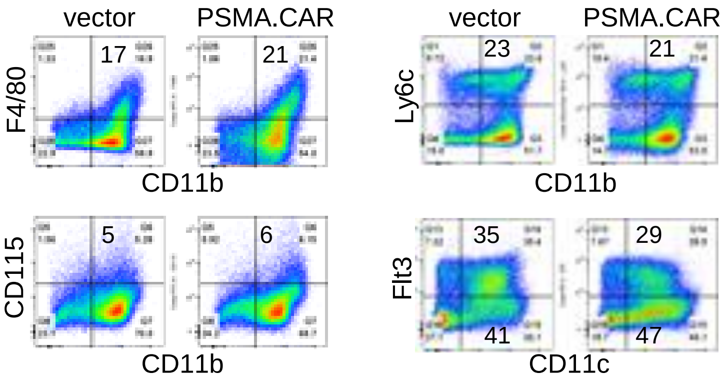

Fig. S7 p50-IMC transduced with MIPuro (vector) or PSMA.CAR10.3 were analyzed for the indicated myeloid surface markers by flow cytometry. These data are from a single experiment.
